# Using Mindfulness to Reduce Anxiety and Depression of Patients With Fever Undergoing Screening in an Isolation Ward During the COVID-19 Outbreak

**DOI:** 10.3389/fpsyg.2021.664964

**Published:** 2021-05-28

**Authors:** Yuping Liu, Sizhu Huyang, Haihong Tan, Yubiao He, Jin Zhou, Xue Li, Man Ye, Jin Huang, Daxing Wu

**Affiliations:** ^1^Department of Infectious Diseases, The Second Xiangya Hospital, Central South University, Changsha, China; ^2^Medical Psychological Center, The Second Xiangya Hospital of Central South University, Changsha, China; ^3^Medical Psychological Institute of Central South University, Changsha, China; ^4^Clinical Nursing Teaching and Research Section, The Second Xiangya Hospital, Central South University, Changsha, China; ^5^National Clinical Research Center for Mental Disorders, Changsha, China

**Keywords:** coronavirus disease 2019, brief mindfulness intervention, isolation ward, anxiety, depression

## Abstract

The coronavirus disease 2019 (COVID-19) continues to spread globally. This infectious disease affects people not only physically but also psychologically. Therefore, an effective psychological intervention program needs to be developed to improve the psychological condition of patients screened for fever during this period. This study aimed to investigate the effect of a brief mindfulness intervention on patients with suspected fever in a screening isolation ward awaiting results of the COVID-19 test. The Faces Scale and the Emotional Thermometer Tool were used to investigate 51 patients who were randomly divided into an intervention group and a control group. All patients completed self-rating questionnaires online at the time they entered the isolation ward and before they were informed of the results. The intervention group listened to the mindfulness audios through hospital broadcasts in the isolation ward before their lunch break and while they slept. Compared with the control group, the intervention group’s life satisfaction score increased (*F* = 4.02, *p* = 0.051) and the emotional thermometer score decreased (*F* = 8.89, *p* = 0.005). The anxiety scores (*F* = 9.63, *p* = 0.003) and the needing help scores decreased significantly (*F* = 4.95, *p* = 0.031). Distress (*F* = 1.41, *p* = 0.241), depression (*F* = 1.93, *p* = 0.171), and anger (*F* = 3.14, *p* = 0.083) also decreased, but did not reach significance. Brief mindfulness interventions can alleviate negative emotions and improve the life satisfaction of patients in the isolation ward who were screened for COVID-19 during the waiting period.

## Introduction

From December 2019, the outbreak of the coronavirus disease 2019 (COVID-19) has had a massive impact on both physical and psychological well-being. Fever, tiredness, and dry cough are the most common symptoms of COVID-19. Most people can recover without special treatment, but they are highly contagious and can be infectious during the incubation period. Thus, the transmission speed of COVID-19 was not fully understood during the initial stage. The National Health Commission of China (NHC) responded swiftly and included COVID-19 in Category B of notifiable diseases, defined by the *Law of the People’s Republic of China on the Prevention and Treatment of Infectious Diseases* (*Revised*; CDC, 2020) on January 20th, 2020. The NHC announced that the country would implement preventive and control measures for Category A of infectious diseases to effectively fight against pneumonia caused by the novel coronavirus. The government has taken several efficient measures to curb the spread of the epidemic, such as halting most businesses and social activities, quarantining measures, assigning designated hospitals for COVID-19 treatment, and building cabin hospitals.

An online survey found that over half of the respondents were psychologically affected and one-third of the respondents showed moderate-to-severe anxiety (Wang et al., 2020c). Several longitude researches in different countries have reported that, during the lockdown, people showed a significant increase in depression, anxiety, and psychological distress, some people even experienced PTSD-related symptoms (Pierce et al., 2020; Planchuelo-Gómez et al., 2020; Roma et al., 2020; Wang et al., 2020b). Moreover, the results of the Di Giuseppe et al. (2020) and Planchuelo-Gómez et al. (2020) suggest that contact with positive cases, lockdown time, the young age, female gender, and consumption of information about COVID-19 were risk factors for psychological symptoms. When people are confined to their homes or some designated places, they spend most of their day watching the news or browsing websites for information about COVID-19 and worrying about their family members who may or may not contract the disease. Contrary to the outbreak of severe acute respiratory syndrome in 2002, online information has replaced newspapers and TV coverage and has become the main source for people to obtain information. However, the information contains not only official reports but also rumors (The Lancet, 2020; Wang et al., 2020c). Meanwhile, self-quarantining can result in people spending too much time on the internet, which leads to social isolation and causes emotional discomfort and psychological stress (Barbisch et al., 2015).

Since the outbreak of COVID-19 in Wuhan, a fever screening system in the isolation ward of a 3A grade hospital has begun to treat and test symptomatic COVID-19 patients. Doctors evaluate the clinical status, survey past, and epidemiology history of outpatients, and then transfer these patients to a fever screening in an isolation ward. Patients stayed in the ward until the test results were obtained. During quarantine, patients suffered from physical discomfort and psychological distress such as feelings of fear, loneliness, terror, and anger (Xiao, 2020). On January 1st, 2020, the NHC issued guiding principles for emergency psychological crisis interventions for the outbreak of COVID-19. They suggested that we do our best to prevent the further spread of COVID-19 and simultaneously pay attention to psychological crisis interventions to reduce the negative impact on people’s psychological well-being and provide specific instructions of psychological intervention to different patients, medical staff, and non-clinical people.

Based on a study of people living in Italy during COVID-19 Pandemic (Conversano et al., 2020b), research indicated that besides social relationship and older age, mindfulness is also an important protective factor against psychological distress. To be specific, mindfulness can help us deal with the stress situation what we are going through, which is based on two primary elements in clinical psychology: (1) people are aware of their present experience and (2) do not judge the present experience and accept the present experience (Keng et al., 2011). In recent years, mindfulness-based interventions have been widely used in clinical patients such as those with cancer, psychological disorders, psychiatric illnesses, and non-clinical patients. Researchers suggest that mindfulness can have a positive influence on psychology, including improving well-being, reducing psychological syndromes, and even modulating behaviors. After mindfulness interventions, people showed a reduction in anxiety levels (Hoge et al., 2013; Würtzen et al., 2013), depression (Deyo et al., 2009; Würtzen et al., 2013), anger, and an increase in forgiving tendencies, life satisfaction, and life equality (Brown and Ryan, 2003). Regardless of short or long-term mindfulness interventions (Shapiro et al., 2006; Lorca et al., 2019), people in the experimental groups showed promising changes during the interventions compared to the control group.

In our research, we aimed to observe the psychological states of patients who were isolated in a ward, promote awareness of COVID-19, and compare the effects of the psychological intervention. We instructed these inpatients to apply mindfulness through standard intervention recording *via* hospital broadcasts instead of face-to-face interactions to minimize the possibility of infection.

## Materials and Methods

### Participants

In this study, we recruited participants from February 1, 2020 to April 30, 2020. The participants were patients with fever who underwent screening in an isolation ward of a 3A grade hospital. The inclusion criteria were patients who (1) had a clear consciousness, (2) were over 18 years of age, (3) had suspected fever or needed to be further diagnosed, (4) were willing to cooperate with the investigation and psychological intervention, and (5) were able to use WeChat and complete questionnaires online. A total of 51 patients were recruited for the study by convenience sampling and were divided into the intervention group (odd day of admission date) and the control group (even day of admission date) according to their admission time. Participation was voluntary and informed consent was obtained. The experimental protocols were approved by the Ethical Committee of the Second Xiangya Hospital of Central South University.

Table 1 presents the demographic information of the intervention group (*n* = 25) and control group (*n* = 26), which included gender, age, education level, marital status, and living situation (with or without family). We also used the Generalized Anxiety Disorder Scale (GAD-7; Spitzer et al., 2006) and the Patient Health Questionnaire (PHQ-9; Kroenke et al., 2001) to evaluate anxiety level and patients’ mental status between the two groups.

**Table 1:** Characteristics of intervention and control patients: mean ± SD.

	Intervention group (*N* = 25)	Control group (*N* = 26)	*χ*^2^/*t*	*p*
Demographics				
Gender (*n*, %)			4.45	0.048^*^
Female	11 (44%)	7 (27%)		
Male	14 (56%)	19 (73%)		
Age (years)	33.74 ± 13.04	35.85 ± 11.91	−0.59	0.557
Highest educational level (*n*, %)			7.87	0.089
Primary school	1 (4%)	3 (12%)		
Junior middle school	3 (12%)	3 (12%)		
Senior middle school	3 (12%)	8 (31%)		
University	16 (64%)	7 (27%)		
Post-graduate degree	2 (8%)	5 (19%)		
Marriage status (*n*, %)			2.69	0.227
Single	12 (48%)	7 (27%)		
Married	12 (48%)	18 (70%)		
Divorced/widowed	1 (4%)	1 (4%)		
Living situation (*n*, %)			0.60	0.499
Alone	6 (24%)	4 (15%)		
With family member	19 (76%)	22 (85%)		
Nervousness about COVID-19	2.56 ± 1.39	2.88 ± 1.11	−0.93	0.359
Panic regarding COVID-19	2.56 ± 1.19	2.85 ± 1.23	−0.85	0.402
GAD-7	4.04 ± 4.01	5.35 ± 3.93	−1.17	0.250
PHQ-9	4.00 ± 3.71	5.35 ± 4.35	−1.19	0.241

SD, standard deviation; *χ*^2^, chi-square test for categorical variables; GAD-7, Generalized Anxiety Disorder Scale; PHQ-9, Patient Health Questionnaire.

* *p* < 0.05.

### Materials

The Frequently Asked Questions about COVID-19 (FAQ-C) was compiled by specialists and professors based on *Public protection and psychological counseling about COVID-19*, published by the Second Xiangya Hospital of Central South University. All the questions were multiple-choice and alternative questions, and were graded out of 100 points.

The Faces Scale (FS; Andrews and Crandall, 1976; Andrews and Stephen, 1976) was utilized. The scale contained eight different cartoon faces, which varied from a very happy face to a very sad face, and aimed to assess satisfaction with recent life (positive and negative feelings).

The Emotional Thermometer Tool (ET; Mitchell et al., 2010a,b) is a visual analog screening tool used to detect emotional disorders in a clinical situation. We adopted the Chinese version of ET (Cheng et al., 2021), which consisted of five items: distress, anxiety, depression, anger, and help (*e.g., In the first four columns, please circle the number that best describes how much emotional upset you have been experiencing in the past week, including today since outbreak of the coronavirus disease 2019. In the last column, please indicate how much you need help for these concerns*). Cronbach’s alpha reliability in this research was 0.78.

The seven-item GAD-7 (Spitzer et al., 2006) is designed to screen for generalized anxiety disorder and evaluate its severity (*e.g., Feeling nervous, anxious or on edge?*). The questionnaire was widely used in clinical practice and the situation of patients was assessed for the past 2 weeks. In this study, Cronbach’s alpha was 0.894.

The PHQ-9 (Kroenke et al., 2001) has diagnostic validity and is efficient in clinical situations. This questionnaire had nine items and only took patients a few minutes to complete to screen for depression in the past 2 weeks (*e.g., Thoughts that you would be better off dead or of hurting yourself in some way?*). Researchers should be aware of people with a score of over five in the case of depression. Cronbach’s alpha reliability of this study was 0.850.

### Procedure

#### Routine Procedure

All participants received routine care. In the isolation ward, we followed the standard operating procedure to allow patients to be hospitalized for treatment. We taught them about sterilization and quarantine measures. In addition, patients took medicines prescribed by their doctors and remained on proper treatment according to their state of illness.

We educated patients about COVID-19 to prevent further spread and promoted awareness of COVID-19, which was referred to as *Public protection and psychological counseling about COVID-19*, published by the Second Xiangya Hospital of Central South University. The control group received routine care and scientific information about COVID-19. Compared to the control group, the intervention group received the same along with psychological intervention.

The patients filled out the questionnaire twice, each time taking about 5 min. First, at the time of admission, and then again before the results from the COVID-19 testing laboratory. The interval between the two questionnaires varies from about 10 to 24 h depending on the time of admission. The COVID-19 test results for all patients were negative.

#### Brief Mindfulness Intervention

The intervention group also received psychological intervention. We built a professional psychological service team that consisted of professors of clinical psychology, head nurses, and experienced core members of our department. During the waiting time, our members monitored patients’ feelings and used an online psychological service platform for one-on-one communication. Meanwhile, we encouraged patients to share about their experience and encouraged them to relieve their psychological burdens with positive speech and behaviors.

We sent light music to participants *via* WeChat; moreover, the ward’s broadcast would play music for 30–60 min during the lunch break and before sleep. The selected music was composed of BANDARI light music and a mindfulness instruction audio. In the 25-min mindfulness instruction audio, the speaker helped the patients pay attention to themselves by using guiding words to focus repeatedly on the breath or other objects, and consciously relaxing all parts of the body in order to concentrate, increase the feeling of the self-body, and focus on the present moment.

### Statistical Analysis

All data were analyzed using SPSS25 and GraphPad Prism8 (GraphPad Software, San Diego, CA, United States). We performed a chi-square test or an independent-samples *t*-test to compare the characteristics of patients in the two groups. The results of GAD-7 and PHQ-9 between groups were compared using independent-sample *t*-tests. First, the results of the FAQ-C, FS, and ET questionnaires before and after the intervention were separately conducted using the independent-samples *t*-test. Second, we performed a two-way repeated measures ANOVA to further compare the effect of a mindfulness intervention on the two groups, with gender as a covariate. Statistical significance was set at *p* < 0.05.

## Results

There were no significant differences between the intervention and control groups in terms of age, education level, marriage, and living situation (Table 1). However, there was a significant difference in gender (*χ*^2^ = −0.59, *p* = 0.048). The scores of the GAD-7 (*t* = −1.17, *p* = 0.250) and PHQ-9 (*t* = −1.19, *p* = 0.241) did not differ significantly between the two groups.

We recorded the baseline and post-intervention scores of FAQ-C, FS, the total score of ET, and five sub-tests scores of ET. The baseline scores between the two groups were not significantly different (Table 2). After the intervention, compared to the control groups, the ET (*t* = 13.08, *p* = 0.001), ET-distress (*t* = 12.71, *p* = 0.001), ET-anxiety (*t* = 8.67, *p* = 0.005), ET-depression (*t* = 5.78, *p* = 0.020), ET-anger (*t* = 9.41, *p* = 0.004), and ET-help (*t* = 5.86, *p* = 0.019) were significantly lower in the intervention group, and FS scores were much higher (*t* = 9.71, *p* = 0.003).

**Table 2:** Independent-samples *t*-test results of pre‐ and post-intervention between two groups^†^.

	Inter. (SD)	Con. (SD)	*t*	*p*
Pre-intervention				
FAQ	85.20 (12.95)	80.77 (15.98)	0.81	0.374
FS	4.92 (1.04)	4.65 (1.50)	1.34	0.253
ET	13.08 (9.40)	16.42 (11.61)	1.08	0.303
Distress	2.80 (2.42)	4.46 (3.24)	3.90	0.054
Anxiety	3.52 (3.08)	4.12 (3.02)	0.14	0.710
Depression	1.40 (1.68)	2.12 (2.74)	0.93	0.340
Anger	1.60 (2.45)	1.73 (2.51)	0.09	0.765
Help	3.76 (3.49)	4.00 (3.81)	0.12	0.728
Post-intervention				
FAQ	86.04 (23.12)	86.54 (13.84)	0.13	0.716
FS	5.56 (0.96)	4.50 (1.42)	9.71	0.003^**^
ET	9.04 (7.63)	18.15 (10.71)	13.08	<0.001^***^
Distress	2.08 (2.18)	4.58 (2.76)	12.71	<0.001^***^
Anxiety	2.32 (2.53)	4.42 (2.64)	8.67	0.005^**^
Depression	1.08 (1.44)	2.50 (2.37)	5.78	0.020^*^
Anger	0.56 (0.92)	2.00 (2.48)	9.41	0.004^**^
Help	3.00 (2.87)	4.96 (3.25)	5.86	0.019^*^

SD, standard deviation.

† Covariance: gender. FAQ, The Frequently Asked Questions about COVID-19 scores; FS, The Faces Scale scores; ET, The Emotional Thermometer Tool scores.

* *p* < 0.05;

** *p* < 0.01;

*** *p* < 0.001.

Although the score of the first test was not significantly different, further analysis is needed to rule out its effect. A two-way repeated measures ANOVA was used to compare the difference in the second-test score between the intervention and control groups controlling for baseline, and gender was also used as a covariate. The results of the analysis, controlling for pre-test scores, are shown in Table 3.

**Table 3:** Results of the FAQ, FS, and ET in the control and intervention group before and after mindfulness intervention with two-way repeated measures ANOVA^†^.

	Effect	*F* ratio	*p*	Partial *η*^2^
FAQ	Group	0.04	0.836	0.001
	Time	1.21	0.277	0.010
	Group × Time	1.60	0.212	0.032
FS	Group	5.74	0.021^*^	0.107
	Time	2.04	0.160	0.041
	Group × Time	4.02	0.051	0.077
ET	Group	5.83	0.020^*^	0.108
	Time	0.90	0.347	0.018
	Group × Time	8.89	0.005^**^	0.156
ET-Distress	Group	9.78	0.003^**^	0.169
	Time	0.05	0.824	0.001
	Group × Time	1.41	0.241	0.029
ET-Anxiety	Group	2.79	0.101	0.055
	Time	2.87	0.097	0.056
	Group × Time	9.63	0.003^**^	0.167
ET-Depression	Group	3.24	0.078	0.063
	Time	0.10	0.756	0.002
	Group × Time	1.93	0.171	0.039
ET-Anger	Group	3.61	0.063	0.070
	Time	0.09	0.766	0.002
	Group × Time	3.14	0.083	0.061
ET-Help	Group	2.13	0.151	0.043
	Time	0.33	0.567	0.007
	Group × Time	4.95	0.031^*^	0.095

FAQ, The Frequently Asked Questions about COVID-19 scores; FS, The Faces Scale scores; ET, The Emotional Thermometer Tool scores.

† Covariance: gender.

* *p* < 0.05;

** *p* < 0.01.

**Figure 1:**
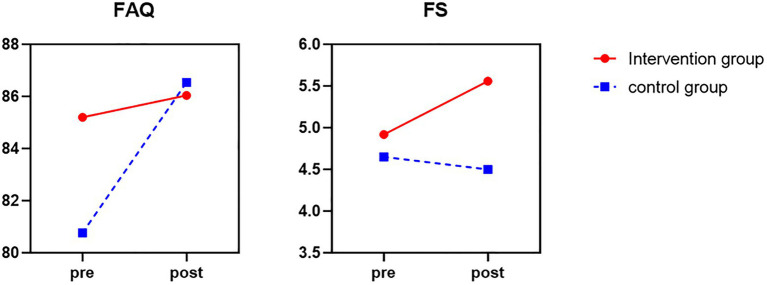
The score of FAQ and FS before and after mindfulness intervention between control group (blue line) and intervention group (red line).

**Figure 2:**
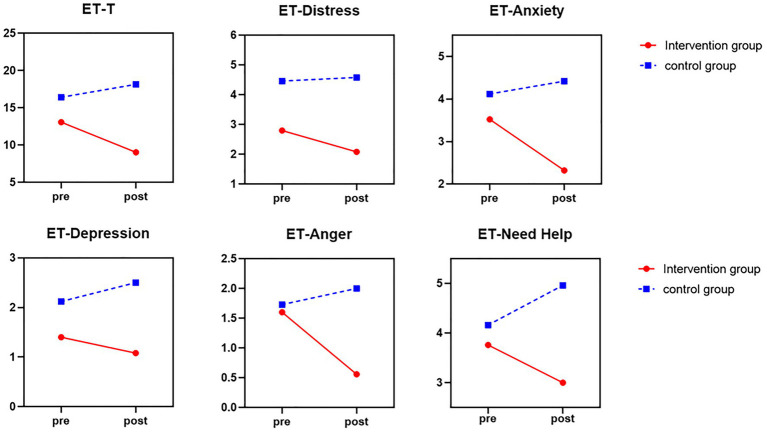
The score of total mean ET and five subscales of ET before and after mindfulness intervention between control group (blue line) and intervention group (red line).

There was a marginally significant Group × Time interaction of total FS score, *F* = 4.02, *p* = 0.051, partial *η*^2^ = 0.032. As can be seen in Figure 1, after brief mindfulness intervention, the score of FS in the intervention group was much lower than the control group. As shown in Figures 1, 2, the scores of the individual ET items, all subtests, including “Distress,” “Anxiety,” “Depression,” “Anger,” and “Need Help,” were reduced in the intervention group, while the scores of the control group were increased. The mean total ET score was much lower (*F* = 8.89, *p* = 0.005, partial *η*^2^ = 0.156) compared to the control group. Meanwhile, the anxiety subscale (*F* = 9.63, *p* = 0.003, partial *η*^2^ = 0.167) and help subscale (*F* = 4.95, *p* = 0.031, partial *η*^2^ = 0.095) were significantly lower in the intervention group than in the control group. However, the distress subscale (*F* = 1.41, *p* = 0.241, partial *η*^2^ = 0.029), depression subscale (*F* = 1.93, *p* = 0.171, partial *η*^2^ = 0.039), and anger subscale scores (*F* = 3.14, *p* = 0.083, partial *η*^2^ = 0.061) were not statistically significant.

## Discussion

Our study focused on whether the psychological states of patients entering the isolation ward were improved after a brief mindfulness intervention. The primary findings suggested that patients in the intervention group showed mood modification after the brief mindfulness-based intervention. This may be related to the increased attention brought about by mindfulness interventions, which enable patients in the intervention group to find ways to cope with and manage stressful emotions, while reducing the perception of negative emotions such as depression and anxiety (Conversano et al., 2020a). Compared to the control group, they felt more satisfied with life in the second test, and the levels of distress, anxiety, depression, anger, and needing help decreased. In contrast, the score of patients in the control group increased during the waiting period. Specifically, there was an increase in FS, and the decrease in mean total ET, anxiety, and ET-anger was significant. Interestingly, the ET-distress scores showed significant main effect of group but no significant interaction, which may be due to the margin significant difference between two groups at baseline. The later studies could add scales measuring psychological distress to control for differences between the two groups to better observe the effect of brief mindfulness interventions on psychological distress. Although some subscale scores were not statistically significant, this result still indicated that brief mindfulness intervention can help patients in an isolation ward to improve their psychological condition and make them feel more positive in the face of uncertain outcomes.

In the last few decades, mindfulness-related interventions have been applied extensively both in China and abroad, especially in clinical settings (Hoge et al., 2013; Würtzen et al., 2013; Liu et al., 2019; Buckner et al., 2020); however, they also affect the non-clinical population (Arch and Craske, 2006; Zhu et al., 2019). Recently, Lorca et al. (2019) suggested that a single-session mindfulness practice using a meditation recording reduced both subjective and objective anxiety in patients undergoing a PET/CT study. With the support of a previous study, mindfulness interventions can significantly improve participants’ positive emotions (Keng et al., 2011), reduce the self-reported level of anxiety and depression (Hoge et al., 2013; Würtzen et al., 2013; Liu et al., 2019; Zhu et al., 2019; Buckner et al., 2020), and even adjust heart rate (Lorca et al., 2019). Our results are consistent with the conclusion that mindfulness interventions can adjust a patient’s mood and may have clinical implications for people in quarantine to reduce psychological stress.

However, some deficiencies in our research could be avoided in further studies. The sample of the present study was small and the gender ration of patients was not equal. The sample can be expanded in future research and the ratio of gender could be balanced as much as possible to exclude the effect of gender on the results. In addition, the current mindfulness-based intervention was conducted during a short period, and only compares the condition of isolated patients when they were admitted in the ward for 24 h before they heard of their results from the laboratory. In the future, researchers should record the periodic changes in psychological conditions and add mindfulness questionnaires to monitor how patients accept mindfulness interventions during the entire quarantine period. In addition, because of the high infectiousness, all interventions and questionnaires were online or through broadcast without face-to-face communication. Our research is primary, but importantly, it may provide some suggestions for improvement in mood and interventions.

## Data Availability Statement

The raw data supporting the conclusions of this article will be made available by the authors, without undue reservation.

## Ethics Statement

The studies involving human participants were reviewed and approved by the Second Xiangya Hospital of Central South University Ethics Committee. The patients/participants provided their written informed consent to participate in this study. Informed consent was obtained from all participants.

## Author Contributions

DW and JH conceived and designed the study. All the authors were involved in the study. YL, SH, and DW performed the analysis and prepared the manuscript. All co-authors contributed substantially to the revision and have approved the final manuscript.

### Conflict of Interest

The authors declare that the research was conducted in the absence of any commercial or financial relationships that could be construed as a potential conflict of interest.

## References

[ref1] Andrews F. M. Crandall R. (1976). The validity of measures of self-reported well-being. Soc. Indic. Res. 3, 1–19. 10.1007/BF00286161

[ref2] Andrews F. M. W. Stephen B. (1976). Social Indicators of Well-Being: Americans’ Perceptions of Life Quality. Boston, MA: Springer.

[ref3] Arch J. J. Craske M. G. (2006). Mechanisms of mindfulness: emotion regulation following a focused breathing induction. Behav. Res. Ther. 44, 1849–1858. 10.1016/j.brat.2005.12.007, PMID: 16460668

[ref4] Barbisch D. Koenig K. L. Shih F. Y. (2015). Is there a case for quarantine? Perspectives from SARS to Ebola. Disaster Med. Public Health Prep. 9, 547–553. 10.1017/dmp.2015.38, PMID: 25797363

[ref5] Brown K. W. Ryan R. M. (2003). The benefits of being present: mindfulness and its role in psychological well-being. J. Pers. Soc. Psychol. 84, 822–848. 10.1037/0022-3514.84.4.822, PMID: 12703651

[ref6] Buckner J. D. Lewis E. M. Abarno C. N. Heimberg R. G. (2020). Mindfulness training for clinically elevated social anxiety: the impact on peak drinking. Addict. Behav. 104:106282. 10.1016/j.addbeh.2019.106282, PMID: 31918168

[ref7] CDC (2020). Law of the People’s Republic of China on the Prevention and Treatment of Infectious Diseases (Revised). Available at: http://www.nhc.gov.cn/jkj/s7916/202001/44a3b8245e8049d2837a4f27529cd386.shtml (Accessed January 20, 2020).

[ref8] Cheng C. Jing Z. Li Z. Jie B. (2021). The Chinese Version of Emotion Temperature. Available at: http://www.psycho-oncology.info/ET_chinese.pdf (Accessed April 12, 2021).

[ref9] Conversano C. Ciacchini R. Orrù G. Di Giuseppe M. Gemignani A. Poli A. (2020a). Mindfulness, compassion, and self-compassion among health care professionals: what’s new? A systematic review. Front. Psychol. 11:1683. 10.3389/fpsyg.2020.01683, PMID: 32849021 PMC7412718

[ref10] Conversano C. Di Giuseppe M. Miccoli M. Ciacchini R. Gemignani A. Orrù G. (2020b). Mindfulness, age and gender as protective factors against psychological distress during COVID-19 pandemic. Front. Psychol. 11:1900. 10.3389/fpsyg.2020.01900, PMID: 33013503 PMC7516078

[ref11] Deyo M. Wilson K. A. Ong J. Koopman C. (2009). Mindfulness and rumination: does mindfulness training lead to reductions in the ruminative thinking associated with depression? Explore 5, 265–271. 10.1016/j.explore.2009.06.005, PMID: 19733812

[ref800] Di Giuseppe M. Perry J. C. Conversano C. Gelo O. C. G. Gennaro A. (2020). Defense mechanisms, gender and adaptiveness in emerging personality disorders in adolescent outpatients. J. Nerv. Ment. Dis. 208, 933–941. 10.1097/NMD.0000000000001230, PMID: 32947450

[ref12] Hoge E. A. Bui E. Marques L. Metcalf C. A. Morris L. K. Robinaugh D. J.. (2013). Randomized controlled trial of mindfulness meditation for generalized anxiety disorder: effects on anxiety and stress reactivity. J. Clin. Psychiatry 74, 786–792. 10.4088/JCP.12m08083, PMID: 23541163 PMC3772979

[ref13] Keng S. L. Smoski M. J. Robins C. J. (2011). Effects of mindfulness on psychological health: a review of empirical studies. Clin. Psychol. Rev. 31, 1041–1056. 10.1016/j.cpr.2011.04.006, PMID: 21802619 PMC3679190

[ref14] Kroenke K. Spitzer R. L. Williams J. B. (2001). The PHQ-9: validity of a brief depression severity measure. J. Gen. Intern. Med. 16, 606–613. 10.1046/j.1525-1497.2001.016009606.x, PMID: 11556941 PMC1495268

[ref15] Liu H. Gao X. Hou Y. (2019). Effects of mindfulness-based stress reduction combined with music therapy on pain, anxiety, and sleep quality in patients with osteosarcoma. Braz. J. Psychiatry 41, 540–545. 10.1590/1516-4446-2018-0346, PMID: 31116262 PMC6899366

[ref16] Lorca A. M. Lorca M. M. Criado J. J. Aguado R. Baños M. C. Z. Armesilla M. D. C. (2019). Using mindfulness to reduce anxiety during PET/CT studies. Mindfulness 10, 1163–1168. 10.1007/s12671-018-1065-2

[ref17] Mitchell A. J. Baker-Glenn E. A. Granger L. Symonds P. (2010a). Can the distress thermometer be improved by additional mood domains? Part I. initial validation of the emotion thermometers tool. Psychooncology 19, 125–133. 10.1002/pon.1523, PMID: 19296462

[ref18] Mitchell A. J. Baker-Glenn E. A. Park B. Granger L. Symonds P. (2010b). Can the distress thermometer be improved by additional mood domains? Part II. What is the optimal combination of emotion thermometers? Psychooncology 19, 134–140. 10.1002/pon.1557, PMID: 19296461

[ref19] Pierce M. Hope H. Ford T. Hatch S. Hotopf M. John A.. (2020). Mental health before and during the COVID-19 pandemic: a longitudinal probability sample survey of the UK population. Lancet Psychiatry 7, 883–892. 10.1016/S2215-0366(20)30308-4, PMID: 32707037 PMC7373389

[ref20] Planchuelo-Gómez Á. Odriozola-González P. Irurtia M. J. de Luis-García R. (2020). Longitudinal evaluation of the psychological impact of the COVID-19 crisis in Spain. J. Affect. Disord. 277, 842–849. 10.1016/j.jad.2020.09.018, PMID: 33065825 PMC7476580

[ref21] Roma P. Monaro M. Colasanti M. Ricci E. Biondi S. Di Domenico A.. (2020). A 2-month follow-up study of psychological distress among Italian people during the COVID-19 lockdown. Int. J. Environ. Res. Public Health 17:8180. 10.3390/ijerph17218180, PMID: 33167479 PMC7663995

[ref22] Shapiro S. L. Carlson L. E. Astin J. A. Freedman B. (2006). Mechanisms of mindfulness. J. Clin. Psychol. 62, 373–386. 10.1002/jclp.20237, PMID: 16385481

[ref23] Spitzer R. L. Kroenke K. Williams J. B. Löwe B. (2006). A brief measure for assessing generalized anxiety disorder: the GAD-7. Arch. Intern. Med. 166, 1092–1097. 10.1001/archinte.166.10.1092, PMID: 16717171

[ref700] The Lancet (2020). COVID-19: fighting panic with information. Lancet 395:537. 10.1016/S0140-6736(20)30379-2, PMID: 32087777 PMC7138040

[ref25] Wang C. Horby P. W. Hayden F. G. Gao G. F. (2020c). A novel coronavirus outbreak of global health concern. Lancet 395, 470–473. 10.1016/S0140-6736(20)30185-9, PMID: 31986257 PMC7135038

[ref26] Wang C. Pan R. Wan X. Tan Y. Xu L. Ho C. S.. (2020a). Immediate psychological responses and associated factors during the initial stage of the 2019 coronavirus disease (COVID-19) epidemic among the general population in China. Int. J. Environ. Res. Public Health 17:1729. 10.3390/ijerph17051729, PMID: 32155789 PMC7084952

[ref27] Wang C. Pan R. Wan X. Tan Y. Xu L. McIntyre R. S.. (2020b). A longitudinal study on the mental health of general population during the COVID-19 epidemic in China. Brain Behav. Immun. 87, 40–48. 10.1016/j.bbi.2020.04.028, PMID: 32298802 PMC7153528

[ref28] Würtzen H. Dalton S. O. Elsass P. Sumbundu A. D. Steding-Jensen M. Karlsen R. V.. (2013). Mindfulness significantly reduces self-reported levels of anxiety and depression: results of a randomised controlled trial among 336 Danish women treated for stage I–III breast cancer. Eur. J. Cancer 49, 1365–1373. 10.1016/j.ejca.2012.10.030, PMID: 23265707

[ref29] Xiao C. (2020). A novel approach of consultation on 2019 novel coronavirus (COVID-19)-related psychological and mental problems: structured letter therapy. Psychiatry Investig. 17, 175–176. 10.30773/pi.2020.0047, PMID: 32093461 PMC7047000

[ref30] Zhu T. Xue J. Montuclard A. Jiang Y. Weng W. Chen S. (2019). Can mindfulness-based training improve positive emotion and cognitive ability in Chinese non-clinical population? A pilot study. Front. Psychol. 10:1549. 10.3389/fpsyg.2019.01549, PMID: 31333552 PMC6619344

